# Microbiome Data Analysis by Symmetric Non-negative Matrix Factorization With Local and Global Regularization

**DOI:** 10.3389/fmolb.2021.643014

**Published:** 2021-04-27

**Authors:** Junmin Zhao, Yuanyuan Ma, Lifang Liu

**Affiliations:** ^1^School of Computer and Data Science, Henan University of Urban Construction, Pingdingshan, China; ^2^School of Computer and Information Engineering, Anyang Normal University, Anyang, China; ^3^School of Education, Anyang Normal University, Anyang, China

**Keywords:** matrix factorization, Laplacian regularization, Vicus graph, microbiome, local structure

## Abstract

A network is an efficient tool to organize complicated data. The Laplacian graph has attracted more and more attention for its good properties and has been applied to many tasks including clustering, feature selection, and so on. Recently, studies have indicated that though the Laplacian graph can capture the global information of data, it lacks the power to capture fine-grained structure inherent in network. In contrast, a Vicus matrix can make full use of local topological information from the data. Given this consideration, in this paper we simultaneously introduce Laplacian and Vicus graphs into a symmetric non-negative matrix factorization framework (LVSNMF) to seek and exploit the global and local structure patterns that inherent in the original data. Extensive experiments are conducted on three real datasets (cancer, cell populations, and microbiome data). The experimental results show the proposed LVSNMF algorithm significantly outperforms other competing algorithms, suggesting its potential in biological data analysis.

## Introduction

With the development of high-throughput metagenomic sequencing and 16S sequencing technologies, more and more biological data have been accumulated. Generally, these sequences have very complicated characteristics, making discoveries and identification of latent relations among samples very daunting. In order to reach a good understanding of the roles that the microbiome plays in the health and disease states of humans, many plans, including the Human Microbiome Plan (HMP) (Turnbaugh et al., 2007), integrative Human Microbiome Plan (iHMP) (The Integrative Hmp (iHMP) Research Network Consortium, 2019), and the Metagenomics of Human Intestinal Tract (MetaHIT) (Qin et al., 2010), have been launched. These actions pave the way for researchers to further explore the complex relationships residing in microbiome data.

Arumugam et al. classified the microbiome into different enterotypes and pointed out the significance of a functional analysis to reveal the interactions among microorganisms (Arumugam et al., 2011; Siezen and Kleerebezem, 2011). Clustering approaches and similarity measurements were applied to elucidate the influence that various factors impose on the identification of enterotypes (Koren et al., 2013). Jiang et al. (2012) proposed a new approach based on non-negative matrix factorization (NMF) to identify the structure and functions of complex microbial communities across environmental samples. Wu et al. (2016) developed a stable NMF method, staNMF, and obtained a novel and concise representation of spatial gene expression patterns. As a clustering technology, NMF has attracted a lot of attention in terms of its good data representation capabilities. In NMF, samples or features can be viewed as the linear additive combination of basis vectors. Meanwhile, the membership label of each sample can be assigned by the corresponding coefficient matrix. When the data have a linear structure, NMF usually achieves better performance. However, in the real world, data points are generally embedded in a non-linear manifold; thus, adopting other ways (such as graphs) to describe latent relationships among data points is a better choice (Kuang et al., 2012). Symmetric non-negative matrix factorization (SNMF) takes a similarity matrix as input and outputs a cluster indicator. In this process, a similarity matrix can be obtained in many ways, such as through a Gaussian kernel, polynomial kernel, linear kernel, and so on. Kuang et al. (2012, 2015) designed an effective SNMF algorithm to model the complex relationships contained in non-linear data that outperforms many NMF-based approaches. Ma et al. (2016a) developed HSNMF, a method that combined SNMF with a second-order graph to explore microbiome data.

Recently, the graph regularization framework has been successfully applied in many fields including bioinformatics, image processing, and text mining, achieving good performance. Cai et al. (2010) proposed the GNMF algorithm to reveal the potential patterns of several datasets. Specifically, GNMF used Laplacian regularization (Lr) to encode the intrinsic geometrical structure presented in the original data. Subsequently, a number of variants based on Lr were generated (He et al., 2015; Ma et al., 2016b, 2017). Although these methods obtained some interesting findings, they may ignore some important aspects. For example, a traditional Laplacian graph captures the global structure of a data matrix, which is insufficient in biology research, where local topologies need to be sought and utilized effectively (Wang et al., 2017). Moreover, recently emerging methods developed to capture local topological information have been shown to obviously outperform global algorithms (Roweis and Saul, 2000; Wu and Schölkopf, 2007; Jiang and Hu, 2014). These methods aim to reconstruct each data point using its local neighbors and are shown to be robust and insensitive to outliers. Vicus, an alternative local spectral matrix, can effectively capture the local geometrical information from neighboring nodes to model biological interactions, and it shares many similar properties with Laplacian matrices; for example, both matrices are symmetric and positive semidefinite (psd), and both have non-negative, real-valued eigenvalues (Wang et al., 2017). Compared with Laplacian graphs, Vicus graphs, which are constructed via local subnetworks, are more robust with respect to noise and can lessen the influence of outliers to some extent. In this paper, we first used similarity graphs to establish the complicated relationships in samples; second, given these graphs, we constructed Laplacian and Vicus spectral matrices; finally, we integrated the Vicus (and/or Laplacian) matrix into an SNMF framework to conduct downstream analysis, such as clustering, visualization, and so on.

**FIGURE 1:**
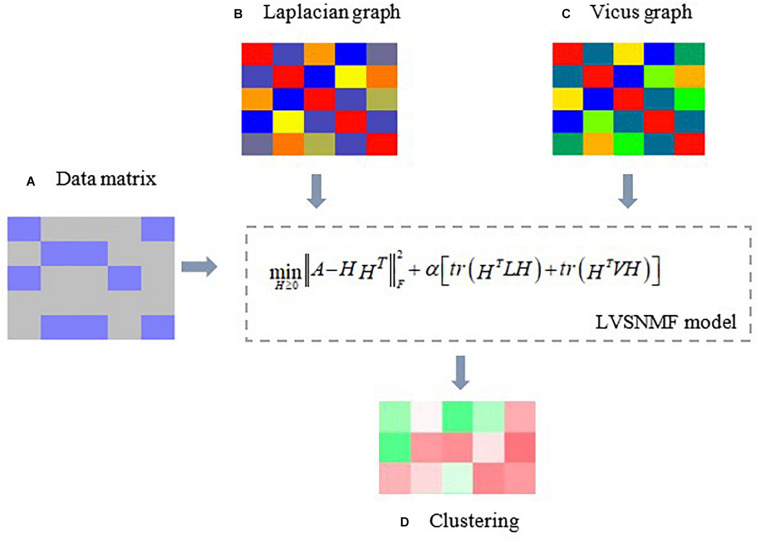
An illustrative example of the proposed LVSNMF algorithm. **(A)** the original data matrix (gene expression matrix, microbiome abundance profile matrix, and so on. **(B)** Laplacian graph used to maintain manifold consistence assumptions. **(C)** A Vicus graph explores the local geometrical structure in the data. Then c and d are introduced into the proposed LVSNMF model, which integrated the global (Laplacian) and local (Vicus) geometrical structure of the original data. **(D)** the clustering result given by LVSNMF.

In view of the above considerations, in this paper we introduce Laplacian and Vicus matrices (Wang et al., 2017) to simultaneously model the global and local structure connections residing within the data and compare their performance with the methods only based on Laplacian or Vicus graphs on several real datasets. Our experiments include tumor classification, microbiome samples identification and so on. The experimental results show that the proposed algorithm outperforms other baseline and competing approaches, which demonstrates its efficiency and effectiveness in microbiome data analysis. Figure 1 gives an illustrative example.

The contribution of this work lies in the fact that (1) an effective clustering algorithm has been proposed and can be easily expanded to other applications and (2) to our knowledge, this is the first attempt to integrate global and local structure information into an SNMF framework to conduct microbiome data analysis. The rest of this paper is organized as follows: in the next section a brief statement of SNMF is given. Then Lr, the formulation of Vicus, and the proposed algorithm are also provided. In section “Results and Discussion”, extensive experiments are conducted, and the experimental results and comparisons analysis are presented. Section “Conclusion” summarizes the conclusions and further research plans.

## Materials and Methods

### Symmetric Non-negative Matrix Factorization

In SNMF, given an *n* × *n* symmetric matrix *A* and a reduced rank *k*, SNMF seeks to find the best factorization so that *A* = *HH*^*T*^, where *H* can be viewed as the cluster indicator. The objective of SNMF can be formalized as follows:

(1) $$O=\min_{H\geq 0}{∥A-H\,H^{T}∥}_{F}^{2}$$

Where $A\in R_{+}^{n\times n}$ with *A* = *A^T^*, $H\in R_{+}^{n\times k}$, and ∥•∥ denotes the c of a matrix. Compared with NMF, SNMF concerns only the factorized similarity matrix *A* and doesn’t consider whether the structure of the data is linear or non-linear. Once *A* is given, SNMF conducts factorization similar to that of NMF. Therefore, SNMF is more suitable to modeling unknown data. For a matrix *A*, *A*_*ij*_, the *ij*-th element of *A*, denotes the similarity score between the *i*th and the *j*th data points. Similarity metrics can take many forms. One common way is to use the Gaussian kernel function to construct a weighted similarity matrix:

(2) $$w_{i\,j}=\exp\left(-\frac{{∥X_{i}-X_{j}∥}_{F}^{2}}{\sigma_{i}\sigma_{j}}\right)\left(i\neq j\right)$$

where, *X_i_* denotes the *i*th data point (sample), σ*_i_* is the Euclidean distance between *X_i_* and its *d*-th neighborhood. We set *d* = 7 as suggested in the literature of Zelnik-Manor and Perona (2005). It is noted that the diagonal elements of similarity *W* are set to be zeros to eliminate self-similarity. Next, we only retain those edges linking nodes with their *p* nearest neighbors *N*(*i*). Thus, the weighted matrix *W* derived from Equation 2 can be rewritten as:

(3) $$w_{i\,j}=\left\{ \begin{array}{c} w_{i\,j},i\,f\,i\in N\,\left(j\right)\,\,o\,r\,\,j\in N\,\left(i\right);\\ 0,o\,t\,h\,e\,r\,w\,i\,s\,e. \end{array}\right.$$

Where *N*(*i*) is the set of neighbors of the *i*th node.

As suggested in [20], *W* can be transformed into the normalized form:

(4) $$A=D^{-1/2}W\,D^{-1/2}$$

here, $D_{i\,i}=\sum_{j=1}^{n}w_{i\,j}$ denotes the degree matrix.

Finally, by multiplication update rules SNMF can obtain the locally optimal solution:

(5) $$h_{i\,k}\leftarrow h_{i\,k}\frac{{\left(A\,H\right)}_{i\,k}}{{\left(H\,H^{T}H\right)}_{i\,k}}$$

### Laplacian Graph

Given *P*, a weighted matrix, let *L* be the Laplacian matrix; *L* can be defined as:

(6) L=D-P

where *D* is a degree matrix and $D_{i\,i}=\sum_{j=1}^{n}d_{i\,j}$. The normalized cut version of *L* can be formalized as:

(7) $$L=I-D^{-1/2}P\,D^{-1/2}$$

where *I* denotes the identity matrix. Note that the general Laplacian matrix (Equation 6) is used in the complete experiments, and the normalized version of *L* is used to be input for spectral methods. In the process of constructing a weighted graph *P*, many similarity functions can be used, such as the inner product function, kernel function, and so on. In our experiments, the Gaussian kernel function (Equation 2) is employed to establish *P*.

### Vicus Graph

As described in Wang et al. (2017), Vicus graphs have the same properties as Laplacian graphs. For example, both matrices are symmetric and positive semidefinite, and the eigenvector corresponding to the smallest eigenvalue 0 of both matrices is the constant vector **1**. Compared with the Laplacian graph, the Vicus graph can capture the local structure within the data. In this subsection, we demonstrate the process of constructing a Vicus graph in detail. Note that the Vicus matrix is constructed based on a sample-sample similarity matrix, which can be computed via any similarity function, such as the Gaussian kernel, the cosine kernel functions, and so on. In this paper, we used the Gaussian kernel for computing the similarities between any two samples. The differences between the Laplacian and the Vicus graphs lies in the fact that the former describes the global structure information inherent in the data whereas the latter captures well the fine-grained topological information present in the biological network. The intuition is that we can use the local connection information from neighboring nodes to make the Vicus matrix more robust with respect to noise. Thus, it helps to lessen the influence of outliers.

Let {x_1_,x_2_,⋯,x_*n*_} be a set of data points. Then v_*i*_ corresponding to *x_i_* denotes the *i*th vertex in a weighted network *P*, and *N*(*i*) represents *x_i_*’s neighbors, not including *x_i_*. Here, the neighborhood size of all nodes is consistent (∥*N*_*i*_∥ = *k*,*i* = 1,2,⋯,*n*).

The main assumption behind Vicus is that the cluster label of the *i*th data point can be inferred from its nearest neighbors *N*(*i*). First, a subnetwork *P_i_* = (*V_i_*,*E_i_*) is extracted such that *V_i_* = *N*(*i*)⋃*x_i_* and *E_i_* represents the edges connecting all points inherent in *V_i_*. Using the label diffusion algorithm (Zhou et al., 2004), a virtual label indicator vector $c_{v_{i}}^{k}$ can be reconstructed as:

(8) $$c_{v_{i}}^{k}=\left(1-\alpha \right)\,{\left(I-\alpha \,S_{i}\right)}^{-1}q_{v_{i}}^{k},1\leq k\leq C$$

Where α ∈ (01) is a constant. In all our experiments, α is set 0.9 as suggested in Wang et al. (2017). *C* is the number of clusters. $q_{v_{i}}^{k}$ is the scaled cluster indicator of *P_i_*.*S_i_* denoting the normalized transition matrix, i.e., $S_{i}\left(u,t\right)=P_{i}\left(u,t\right)\,/\,\sum_{l=1}^{K+1}P_{i}\left(u,l\right)$. $c_{v_{i}}^{k}$ is a vector of *K* + 1 elements; here ${\bar{q}}_{i}^{k}=c_{v_{i}}^{k}\left[K+1\right]$ is the estimate of how likely it is that node *i* belongs to the *k*th cluster. The goal is maximal concordance between ${\bar{q}}_{i}^{k}$ and $q_{i}^{k}$. Let β_*i*_ ∈ *R*^*K* + 1^ be the *i*th row of the matrix (1−α)(*I*−αS_*i*_)^−1^, representing label propagation at its terminal state. We set ${\bar{q}}_{i}^{k}=\beta_{i}q_{v_{i}}^{k}$. Thus, ${\bar{q}}_{i}^{k}$ can be approximated by

(9) $${\bar{q}}_{i}^{k}\approx \frac{\beta_{i}\left[1:K\right]q_{N\,\left(i\right)}^{k}}{1-\beta_{i}\left[K+1\right]}$$

Where β_*i*_[1:*K*] is the first *K* elements of β_*i*_ and β_*i*_[*K* + 1] denotes the (*K* + 1)th element in β_*i*_.

Next, we use matrix *B* to represent the linear relationship ${\bar{q}}^{k}\approx B\,q^{k},k=1,2,\cdots ,C$:

(10) $$B_{i\,j}=\left\{ \begin{array}{c} \frac{\beta_{i}\left[j\right]}{1-\beta_{i}\left[K+1\right]}\,i\,f\,{\,x}_{j}\in N\,\left(i\right)\,\,a\,n\,d\,x_{j}\,i\,s\,\,t\,h\,e\,\,j-t\,h\,\,e\,l\,e\,m\,e\,n\,t\,\,i\,n\,\,N\,\left(i\right);\\ 0\;o\,t\,h\,e\,r\,w\,i\,s\,e \end{array}\right.$$

In order to minimize the difference between ${\bar{q}}^{k}$ and q^*k*^, we can adopt a simple objective as listed below:

(11) $$\begin{array}{c} \sum_{i=1}^{n}\sum_{k=1}^{C}{\left({\bar{q}}_{i}^{k}-q_{i}^{k}\right)}^{2}=\sum_{k=1}^{C}{∥{\bar{q}}^{k}-q^{k}∥}^{2}\approx \sum_{k=1}^{C}{∥q^{k}-B\,q^{k}∥}^{2}\\ =T\,r\,\left(Q^{T}{\left(I-B\right)}^{T}\left(I-B\right)\,Q\right) \end{array}$$

Here, Tr(•) denotes the trace of a matrix. Setting *V* = (*I*−*B*)^*T*^(*I*−*B*), we thus obtain the Vicus matrix. Similarly to the traditional spectral clustering formulation, by performing eigen-decomposition of *V* we can obtain clustering results. In this paper, we use *V* as constraint term to preserve the local manifold structure of the data.

Note that in order to better reveal the complicated relationships among microbiome samples, we simultaneously introduce the Laplacian and Vicus spectral matrices into the objective function of SNMF. The details are given below, in section “Symmetric Non-negative Matrix Factorization Based on Laplacian and Vicus Regularization.”

### Symmetric Non-negative Matrix Factorization Based on Laplacian and Vicus Regularization

Based on analysis above, we combine Laplacian and Vicus matrices into the Symmetric Non-negative Factorization framework to explore the global and local structure inherent in the data. The proposed algorithm, namely LVSNMF, takes full advantage of the global and local consistency of the data to model complex relationships between different samples. The final objective can be defined simply as:

(12) $$O=\min_{H\geq 0}{∥A-HH^{T}∥}_{F}^{2}+\alpha \,\left[t\,r\,\left(H^{T}L\,H\right)+t\,r\,\left(H^{T}V\,H\right)\right]$$

Where α is a regularization parameter and used to balance the trade-off between matrix reconstruction errors and spatial structure preservation. The second term in Equation 12 simultaneously takes into account the global information (Laplacian) and local structure (Vicus) of the data.

To minimize the objective of LVSNMF, we use multiplicative update rules to solve the optimal problem. The updating formula of *H* can be obtained as follows:

(13) $$h_{i\,k}\leftarrow h_{i\,k}\frac{{\left(A\,H\right)}_{i\,k}+\alpha \,{\left[\left(P+V^{-}\right)\,H\right]}_{i\,k}}{{\left(H\,H^{T}H\right)}_{i\,k}+\alpha \,{\left[\left(D+V^{+}\right)\,H\right]}_{i\,k}}$$

Here, *V* = V^+^ − V^−^.

LVSNMF integrates the global and local similarity information inherent in the data, and it can therefore obtain better performance than just using the Laplacian graph or other methods based on local diffusion information from neighboring nodes.

### Evaluation Metrics

The proposed LVSNMF and competing algorithms are evaluated by comparing the generated labels of all samples with the ground truth contained in the datasets. Two common cluster metrics, accuracy (AC) and normalized mutual information (NMI), are used to evaluate the performance of the proposed LVSNMF algorithm. Generally, the higher AC and NMI values achieved, the better the clustering quality is. More detailed information on these two metrics can be found in Xu et al. (2003).

### Datasets

Three datasets are used in our experiments. The first one is a cancer dataset from TCGA, the second is a pollen dataset, and the last is a human microbiome dataset from HMP^1^. The important statistics of these three datasets are summarized in Table 1.

**TABLE 1:** Statistics of the two datasets.

Dataset	Number of samples	Number of features	Number of clusters
Lung cancer	203	3,312	5
Pollen	249	14,805	11
HMP	637	710	7

Lung cancer: This is a benchmark dataset including five cancer subtypes. It can be downloaded from https://jundongl.github.io/scikit-feature/datasets.html.

Pollen: these data consist of 11 cell populations, containing neural cells and blood cells. These data are obtained directly from Pollen et al. (2014).

Human microbiome data: these data consist of 637 samples drawn from seven body sites: one gut (stool), one vagina (posterior fornix), one nasal (anterior nares), one skin site (retroauricular crease), and three oral sites (supragingival plaque, tongue dorsum, and buccal mucosa). Each sample consists of 710 microorganisms. The relative abundance of each at species level wase estimated by MetaphlAn. All the data can be downloaded from the HMP website.

## Results and Discussion

### Experimental Results

In this section, we conduct extensive experiments on these three datasets. The experimental results are shown in Table 2. From this table, we can see that LVSNMF outperforms the second best algorithm at 0.49/1.93% points in terms of AC/NMI on the Lung dataset, 3.22/2.17% points on the Pollen dataset, and the 2.09/0.61% points on HMP dataset.

**TABLE 2:** The best performance in three real datasets.

	Accuracy (%)			Normalized mutual information (%)		
		
	Lung	Pollen	HMP	Lung	Pollen	HMP
SNMF	83.74	84.66	87.84	67.51	86.49	84.52
SNMF + Laplacian	90.64	85.94	88.27	70.03	87.33	84.46
SNMF + Vicus	90.15	85.60	88.49	71.26	87.12	84.95
LVSNMF	91.13	89.16	90.58	72.96	89.50	85.56

The results in Table 2 are obtained when *p* = 12 and *k* = 15 across all three datasets. Here, *p* denotes the number of neighbors in constructing similarity matrix *A*, *k* is the number of local neighbors in constructing a Vicus matrix. For other values, LVSNMF still outperforms these competing algorithms in most cases. Note that in our experiments NNDSVD (Boutsidis and Gallopoulos, 2008) is utilized to enhance the initiation of SNMF-based algorithms, which result in rapid reduction of reconstruction errors.

### Parameter Analysis

In the proposed LVSNMF method, there is one essential parameter: the regularization parameter α. In fact, Laplacian and Vicus matrices should have different weight parameters. In this study, we set them to be equal for convenience. Parameter α reflects the extent to which we want to exert punishment for violating the manifold consistency hypothesis.

**FIGURE 2:**
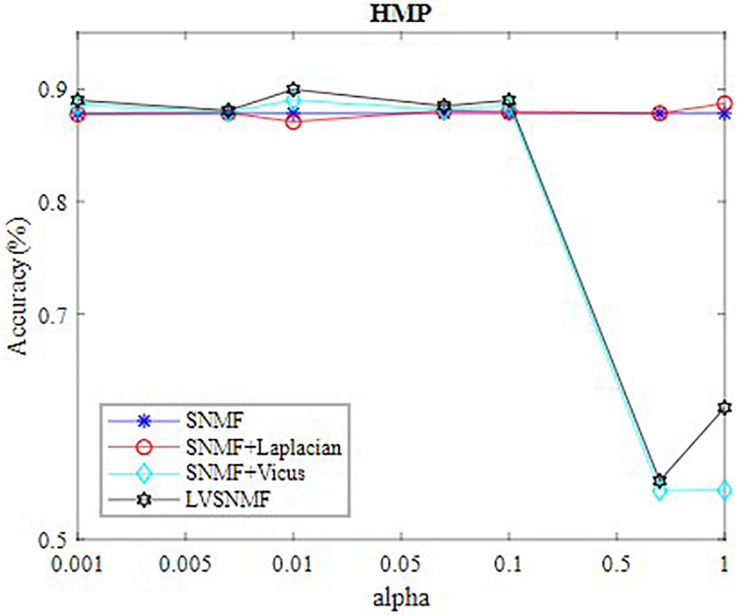
The best performance of LVSNMF as αincreases.

In our experiments, the values of α are set to be in the range (0.001 0.005 0.01 0.05 0.1 0.5 1 10). Table 2 reports the best performance of all algorithms on three datasets. In order to intuitively observe performance of LVSNMF, we draw curve of LVSNMF vs.α on HMP dataset. Figure 2 gives the performance curve of LVSNMF as α increases.

### Comparison and Discussion

In order to further demonstrate the effectiveness of LVSNMF, we compare the performance of LVSNMF with three other algorithms on the HMP dataset when α varies in the range (0.001 0.005 0.01 0.05 0.1 0.5 1). As shown in Figure 1, in the interval (0.001, 0.1), LVSNMF achieves consistently good performance as α increases. When α, LVSNMF obtains the best performance (90.58%/85.56% in terms of AC/NMI), and performs slightly better than the second best algorithm. One possible reason is that samples from HMP are noiseless and the Laplacian has successfully captured sufficient structure information such that the performance of LVSNMF might not be obvious.

On the other hand, on the pollen dataset the proposed LVSNMF algorithm significantly outperforms other competing algorithms (as Table 2 shows). This suggests that LVSNMF has the ability to seek and find the latent structure inherent in the data.

**FIGURE 3:**
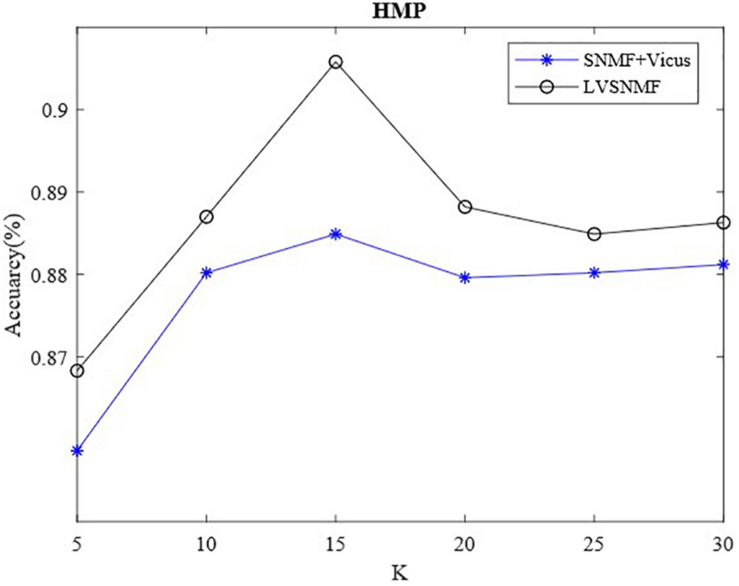
The performance of LVSNMF varies as *K* increases.

Vicus has an important hyperparameter, the number of neighbors *K*. To validate the influence of *K* on the performance of LVSNMF, we also conduct additional experiments on the HMP dataset. Figure 3 shows that the performance of LVSNMF varies with *K* (α = 0.01).

As shown in Figure 3, two algorithms based on Vicus regularization have consistently good performance as *K* varies within the interval (10, 30), especially when *K* equals 15; then they obtain the best performance. This suggests that the proposed LVSNMF is robust with respect to the number of neighbors *K*. Unlike the SNMF + Vicus algorithm, LVSNMF also takes into account the global information of the data; therefore, it can achieve the better performance in most cases.

In summary, the proposed LVSNMF method can adequately capture the global and local structure of the data. The experimental results on three real datasets demonstrate its efficiency and effectiveness.

## Conclusion

In this paper, we propose a novel approach, called LVSNMF, to conduct microbiome data analysis. In LVSNMF, the global and local structure similarities are encoded in Laplacian and Vicus matrices, respectively. Extensive experiments are executed on three datasets, and the results show that the proposed LVSNMF algorithm significantly outperforms other baseline or state-of-the-art methods, which demonstrate its efficiency and effectiveness on microbiome data analysis.

Although LVSNMF achieves good performance, it concerns only sample clustering. In the real world, the relationship among microbes is often subtle and complicated. Therefore, modeling microbial interactions is important to dissect the mechanism behind diseases related to the microbiome. In the future, we will develop new methods to construct microbial interaction networks and seek appropriate ways to describe the functional or genetic similarities among microbes, such as phylogenetic trees, metabolic abilities, and so on.

## Data Availability Statement

Publicly available datasets were analyzed in this study. This data can be found here: https://github.com/chonghua-1983/LVSNMF.

## Author Contributions

JZ and YM wrote the manuscript, designed the algorithms, and conducted all the experiments. YM developed the concept for the structure and content of the manuscript. LL critically revised the final manuscript. All authors reviewed and approved the final version of the manuscript.

## Conflict of Interest

The authors declare that the research was conducted in the absence of any commercial or financial relationships that could be construed as a potential conflict of interest.

## Footnotes

1 http://hmpdacc.org/
